# Hypotension during hip fracture surgery and postoperative morbidity

**DOI:** 10.1007/s11845-020-02175-w

**Published:** 2020-02-13

**Authors:** Gabriel Beecham, Rachael Cusack, Sebastian Vencken, Grace Crilly, Donal J. Buggy

**Affiliations:** 1 Department of Anaesthesiology & Perioperative Medicine, Mater Misericordiae University Hospital, Dublin, Ireland and School of Medicine, University College Dublin, Dublin, Ireland; 2 Clinical Research Centre, School of Medicine, University College Dublin, Dublin, Ireland; 3 Department of Outcomes Research, Cleveland Clinic, Cleveland, OH USA

**Keywords:** Anaesthesia, general, Anaesthesia, spinal, Hip fracture, outcome, Hip fracture, surgery

## Abstract

**Background:**

Hip fracture is a growing healthcare challenge, with 6–8% 30-day mortality and 20–30% of patients incurring major morbidity, including impaired mobilisation and ability to live independently. While observational studies have shown no benefit of general versus spinal anaesthesia on 30-day mortality, intraoperative hypotension during hip fracture surgery is associated with increased 30-day mortality regardless of anaesthetic technique. Although a recent trial on younger patients demonstrated reduced postoperative complications by maintaining intraoperative arterial blood pressure close to preoperative baseline, there are no data correlating intraoperative hypotension during hip fracture surgery with postoperative morbidity.

**Objective:**

We evaluated the hypothesis that duration and severity of intraoperative hypotension during hip fracture surgery is associated with increased postoperative morbidity.

**Methods:**

A retrospective analysis was carried out on *n* = 52 patients undergoing hip fracture surgery between January and June 2017. Measurements of patients’ intraoperative systolic arterial pressure (SAP) and mean arterial pressure (MAP) during anaesthesia, logged electronically through an anaesthesia information management system, were reviewed. We calculated the total duration of time where SAP or MAP were below pre-defined thresholds for hypotension (MAP < 75 mmHg, MAP < 55 mmHg, SAP ≤ 80% admission baseline or SAP ≤ 80% pre-induction baseline). Univariate and bivariate descriptive statistics were generated for all relevant variables. With multivariable regression models containing known predictors, cumulative duration of hypotension was correlated with postoperative comorbidities as quantified by the Clavien-Dindo and Comprehensive Complication Indices.

**Results:**

Mean age (± SD) was 78 ± 13 years, 75% were female, 87% were ASA II or III and 60% underwent spinal anaesthesia. Mean Comprehensive Complication Index was 20.4 ± 19.2. Lowest absolute SAP and MAP values were 82 ± 18 mmHg and 55 ± 12 mmHg respectively. Cumulative time of SAP < 80% pre-induction value adjusted to gender, age and the Charlson Comorbidity Index was associated with progression to a higher Clavien-Dindo classification (odds ratio 1.020 per additional minute; 95% CI 1.008–1.035; *P* = 0.003).

**Conclusions:**

In this exploratory retrospective analysis, the cumulative time of hypotension during hip fracture surgery correlated with extensive postoperative morbidity when adjusting to other known predictors. Intraoperative cumulative time of hypotension may be a good candidate for larger prediction studies as a predictor of postoperative complications. A randomised controlled trial evaluating the effect of actively minimising intraoperative hypotension on postoperative morbidity in hip fracture patients seems warranted.

## Introduction

Hip fracture is a common injury, associated with increased risk of death and major morbidity. Three thousand seven hundred fifty-one patients were treated for hip fracture in Ireland in 2018 [1]. The problem is recognised internationally, with 65,000 cases in the UK in 2016 (costing its National Health Service over £1 billion) and 321,708 cases in the USA in 2014 [2, 3]. There were 2.7 million fragility fractures in 2017 across France, Germany, Italy, Spain, the UK and Sweden, costing €37 billion and with this cost estimated to increase to €47 billion by 2030 [4]. The average age of hip fracture patients is 79 years for men and 81 years for women, with 75% of patients being female. More than half of patients presenting with hip fracture in Ireland and the UK are class III on the American Society of Anaesthesiologists (ASA) physical status classification; 7% are ASA IV, which is associated with a 30-day mortality of 20% [1, 5].

Internationally, the 30-day mortality following hip fracture surgery is 6–8%, a figure which is independent of anaesthetic technique [6]. Hip fracture is one of the commonest reasons for admission of elderly patients to hospital, and compared with elective total hip replacement, it is associated with significant postoperative morbidity including myocardial infarction, cerebrovascular injury, heart failure, renal failure and sepsis [7]. Patients suffering hip fractures have been demonstrated to have a high burden of preoperative comorbidity, increasing the risk of major postoperative complications [8].

Early surgical repair (within 24 h) has been shown to reduce mortality and morbidity in a population-based cohort study [9]. While regional anaesthesia has been hypothesised to be beneficial, recent meta-analyses of observational studies have not demonstrated any benefit of regional versus general anaesthesia on 30-day mortality, although neuraxial techniques appear to be associated with reduced length of hospitalisation and reduced in-hospital mortality [10, 11].

Hypotension occurs commonly during hip fracture repair, especially in the case of older patients with greater numbers of comorbidities [12]. A prospective observational study showed that 89% of patients experienced intraoperative hypotension during hip fracture surgery [5]. One recent randomised controlled trial in younger patients undergoing major non-cardiac surgery showed that the risk of postoperative complications was reduced if the systolic arterial pressure (SAP) was maintained at no more than 10% below the preoperative baseline, using individualised blood pressure management strategies [13]. Prospective hip fracture data from the UK has demonstrated an association between lowest absolute intraoperative blood pressure values and increased 5-day and 30-day mortality [14]. Other data in various surgical populations shows an association between postoperative organ dysfunction and increasing duration and extent of intraoperative hypotension [15].

At present, there are no data correlating the cumulative duration of intraoperative hypotension with postoperative morbidity in patients undergoing hip fracture repair. In this observational study, we tested the hypothesis that duration and severity of intraoperative hypotension are predictors of postoperative morbidity in elderly patients undergoing hip fracture surgery.

## Methods

### Chart review and quantification of baseline comorbidity

We conducted a retrospective observational cohort analysis of all patients who underwent surgery for hip fracture repair in the Mater Misericordiae University Hospital, Dublin, Ireland, between January and June 2017 inclusively. Patients were identified for inclusion in the study through the Hospital In-Patient Enquiry health information system, which generates a basic summary of administrative, clinical and demographic data whenever a patient is discharged from hospital or dies in hospital in Ireland. Patients were therefore included whether they presented through the emergency department from home or were in-patients with other diagnoses who sustained hip fractures in hospital, as well as those transferred from nursing homes or long-term care facilities. Some patients were transferred from other healthcare facilities without a trauma orthopaedic service. All patient clinical data were collected and stored on secured hospital computers with individual password protection for each investigator. Institutional review board approval was obtained for this retrospective analysis.

Clinical records were retrieved for the identified patients, and were analysed for demographic and clinical data including age, gender, preoperative comorbidities, ASA status, length of stay in hospital, comorbidities during the first 30 postoperative days, use of general or spinal anaesthesia and use of non-invasive or invasive blood pressure monitoring. We described the cumulative preoperative comorbidity of our cohort using the Charlson Comorbidity Index; this internationally validated weighted scoring system stratifies patients according to expected in-hospital mortality based on their comorbid condition, assigning expected 1-year mortality rates accounting for number and severity of comorbidities [13]. Data were then correlated to intraoperative events stored electronically and accessed through Centricity High Acuity Anaesthesia (GE Healthcare, Chicago, IL, USA), which is the anaesthesia information management system (AIMS) used in our institution to automatically record intraoperative events and measurements of patient vital signs throughout anaesthesia and recovery.

### Assessing duration of hypotension

Blood pressure measurements were retrieved and analysed for each case from the time of induction of anaesthesia until the time the patient was transferred to the postanaesthesia care unit. We tested four different definitions for intraoperative hypotension:

1. Cumulative time during which SAP was ≤ 80% of the initial measurement taken when the patient was clerked into hospital with a diagnosis of hip fracture;
2. Cumulative time during which SAP was ≤ 80% of the last SAP measurement recorded prior to induction of anaesthesia;
3. Cumulative time during which mean arterial pressure (MAP) was < 75 mmHg; and
4. Cumulative time during which MAP was < 55 mmHg.

Where invasive arterial pressure monitoring was used, recordings had been logged by the AIMS every minute, and where non-invasive monitoring was used, recordings had been logged as per the clinical judgement of the anaesthesiologist caring for the patient, usually every 3 to 5 min. For each of our definitions for intraoperative hypotension, we defined duration of hypotension as the time in minutes between successive blood pressure measurements during anaesthesia where the patient’s most recent recorded blood pressure met the respective criteria. We deemed that a new episode of hypotension started whenever a hypotensive blood pressure measurement was recorded, and that the episode continued until a subsequent normotensive measurement was recorded. We summated these episodes to calculate a cumulative duration of hypotension. This process was repeated four times for each patient’s dataset, applying in turn each definition for intraoperative hypotension.

### Quantification of postoperative morbidity

The patients’ clinical records were analysed to ascertain the incidence of complications postoperatively during the first 30 postoperative days, or until discharge from hospital or death if this occurred sooner. We used a postoperative complications survey (summarised in Table 4), modified from that described by Bennett-Guerrero et al. [16]. Complications of all major organ systems were included: cardiac arrhythmia, myocardial infarction, sepsis, respiratory compromise, thromboembolic events, cerebrovascular events, new cognitive impairment, confusion, coma, renal impairment, liver dysfunction, need for transfusion, need for intensive care, wound dehiscence, pain limiting early postoperative mobilisation, and death.

Postoperative morbidity data was expressed in terms of the Clavien-Dindo classification and Comprehensive Complication Index. The Clavien-Dindo classification qualitatively ranks the severity of a postoperative surgical complication according to the extent of corrective therapy required [15]. We recorded the highest Clavien-Dindo classification associated with each patient based on the single most severe complication they encountered during the first 30 postoperative days. The Comprehensive Complication Index is based on the Clavien-Dindo classification and allows a patient’s overall morbidity to be quantified on a scale from 0 to 100 by integrating multiple weighted Clavien-Dindo classifications for different complications into a composite number [16].

### Statistical analysis

The dependent variables chosen for the prediction models were the Comprehensive Complication Index, which was treated as a continuous outcome variable, and the Clavien-Dindo index, which was treated as an ordinal outcome variable. Because of the limited number of cases per level, particularly at the higher end of the Clavien-Dindo index, the levels 3a, 3b, 4a, 4b and 5 were collapsed to a new level “≥ 3”.

Univariate summary statistics were calculated for all independent and dependent variables.

The association between hypotension and postoperative complications were analysed by multivariable regression. As dependent variables, the collapsed Clavien-Dindo classification and the Comprehensive Complication Index were chosen. The four previously mentioned cumulative time of hypotension variables of interest were included in analysis as continuous independent variables. Additional independent variables were also included due to their previously established association with postoperative complications:

- Age at surgery [17, 18];
- Gender [18, 19];
- Preoperative morbidities as composed in the Charlson comorbidity index *or* the ASA status [18, 20].

To analyse the association between independent variables and postoperative complications, proportional odds models were chosen for the Clavien-Dindo index. The proportional odds assumption of the models was tested using a visual method described by Harrell and the likelihood ratio statistic [21, 22]. Linear models were chosen for the Comprehensive Complication Index. Relative parsimony between base and experimental models was determined using the corrected Akaike’s Information Criterion (AIC_C_) [23, 24]. Likelihood ratio tests were used to compare goodness-of-fit between models. *P* value of 0.05 or less was taken to indicate statistical significance for hypothesis testing. Statistical analyses were performed using the statistical software package R (R Development Core Team, Vienna, Austria) [25].

## Results

### Univariate and bivariate descriptive statistics

*N* = 65 eligible patients were identified for inclusion in the study. Of these, *n* = 13 patients were excluded due to intraoperative data on the AIMS being unavailable or incomplete, due to their surgery not having taken place within the study period, or due to the patients not having proceeded to surgery. This left *n* = 52 patients included in the full analysis. The characteristics of the patient cohort, together with duration of hypotension using our four definitions as well as lowest absolute values for SAP and MAP, as well as postoperative Clavien-Dindo classifications and Comprehensive Complication Index, are summarised in Table 1. Eighty-eight percent of patients had an ASA class of II or III, and 40% underwent general anaesthesia. Of those who received a spinal anaesthetic, 40% had invasive blood pressure monitoring. Comprehensive Complication Index ranged from 0 (no complications) to 100 (death), with a mean average score of 20.4 ± 19.2. Two patients had complications ranked as 4 on the Clavien-Dindo classification, meaning they suffered single or multiple organ failure subsequent to their operation, and one had a complication ranked as 5 (death).

**Table 1:** Univariate patient characteristics and durations of hypotension for the study cohort

Age, years	Mean 77.9, standard deviation ± 12.9
Female gender	*n* = 39, 75% of total
Length of stay in hospital, days	*n* = 28, range 3–122
ASA physical status classification	(% of total)
1	*n* = 4 (8%)
2	*n* = 20 (38%)
3	*n* = 24 (46%)
4	*n* = 1 (2%)
Data not available	*n* = 3 (3%)
Charlson Comorbidity Index	Mean 5.712, range 0–11
Documented surgical procedure	(% of total)
Open reduction of hip with internal fixation	*n* = 8 (15%)
Hemiarthroplasty of hip	*n* = 23 (44%)
Dynamic hip screw	*n* = 8 (15%)
Gamma nail	*n* = 8 (15%)
Total arthroplasty of hip	*n* = 4 (8%)
Revision of total arthroplasty of hip	*n* = 1 (2%)
Anaesthetic technique	(% of total)
SA, NIBP monitoring	*n* = 19 (37%)
SA, arterial line	*n* = 12 (23%)
GA, NIBP monitoring	*n* = 4 (8%)
GA, arterial line	*n* = 17 (33%)
Data not available	*n* = 4 (8%)
Vasopressor boluses administered, number	(% of total)
0	*n* = 13 (25%)
1	*n* = 22 (42%)
2	*n* = 11 (21%)
3	*n* = 5 (10%)
4	*n* = 1 (2%)
Duration of hypotension, minutes	Mean (range)
MAP < 55 mmHg	102.02 (0–362)
MAP < 75 mmHg	83.63 (0–344)
SAP < 80% baseline on hospital admission	69.76 (0–463)
SAP < 80% baseline prior to induction of anaesthesia	63.43 (0–322)
Lowest absolute blood pressure, mmHg	Mean (range)
SAP	82.2 (51–122)
MAP	55.02 (33–92)
Highest single Clavien-Dindo classification	
0	11 (21%)
1	11 (21%)
2	20 (38%)
3	7 (13%)
4	2 (4%)
5	1 (2%)
Comprehensive Complication Index	Mean 20.4, standard deviation ± 19.2, range 0–100

*SA* spinal anaesthesia, *GA* general anaesthesia, *NIBP* non-invasive blood pressure, *ORIF* open reduction with internal fixation

**Fig. 1:**
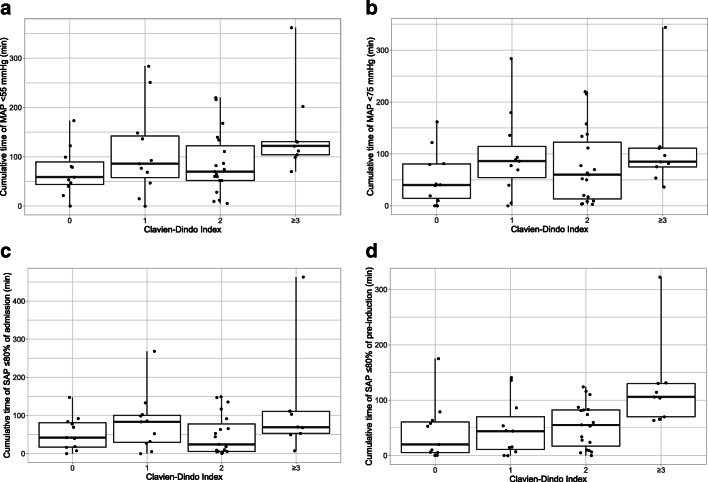
Descriptive bivariate analysis of hypotension variables against Clavien-Dindo index. **a** Time of MAP < 55 mmHg, **b** time of MAP < 75 mmHg, **c** time of SAP < 80% admission baseline, **d** time of SAP < 80% pre-induction baseline. In box plots, the black horizontal bar represents the median, the box represents the interquartile range, the whiskers represent the range and the points the individual data

Bivariate associations of the four cumulative time of hypotension variables against the Clavien-Dindo classification are shown in Fig. 1. The clearest association is the increasing trend between cumulative time of SAP < 80% pre-induction baseline and the Clavien-Dindo classification.

**Fig. 2:**
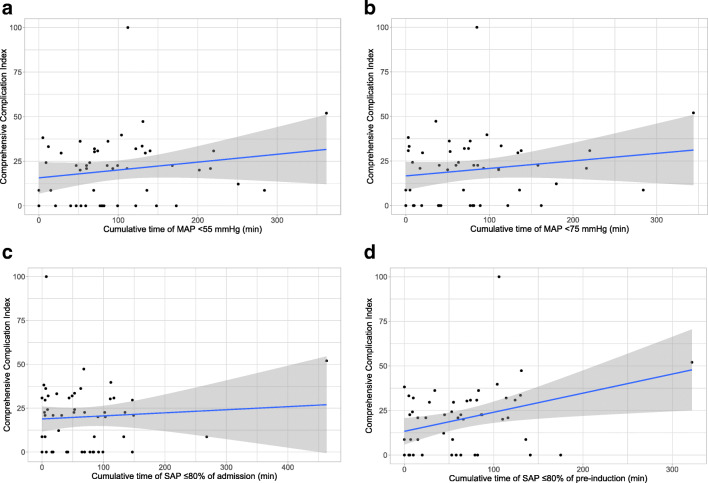
Descriptive bivariate analysis of hypotension variables against the Comprehensive Complication index at 30 days. **a** Time of MAP < 55 mmHg, **b** time of MAP < 75 mmHg, **c** time of SAP < 80% admission baseline, **d** time of SAP < 80% pre-induction baseline. In the scatter plots, line represents the linear correlation as fitted by ordinary least-squares and the shaded area represents the standard error of this fit

Bivariate associations of the four cumulative time of hypotension variables against the Clavien-Dindo classification are shown in Fig. 2. It is difficult to discern any trends between Comprehensive Complication Index and the cumulative time of hypotension variables, except for cumulative time of SAP < 80% pre-induction value, which showed a possible increasing trend.

### Multivariable analysis with the Clavien-Dindo index as outcome

Multivariable regression analysis with the Clavien-Dindo index as dependent variable was performed. Four covariates for hypotension as listed in the “Methods” section were tested for their association with the Clavien-Dindo index, adjusted for base predictors (age, gender and Charlson Comorbidity score). The best fitting model included cumulative time of SAP < 80% pre-induction value as the covariate for hypotension (*P* < 0.001 compared to other models). Adding time SAP < 80% pre-induction value to the model also significantly improved the goodness-of-fit over the base model that did not contain this covariate (*P* < 0.001), while also producing a more parsimonious model based on a lower AIC_C_ over the base model.

As the Charlson Comorbidity Index and ASA classification have previously been shown to correlate with higher morbidity after hip fracture surgery [26], they were both tested as covariates for preoperative comorbidities by comparing these in separate models. The choice of covariate for preoperative comorbidity did not result in a significant improvement of goodness-of-fit of one base model over the other (*P* = 0.186). Due to the fewer degrees of freedom, the model with the Charlson Comorbidity index was more parsimonious.

The odds ratio of progressing to a higher Clavien-Dindo classification for a 1-min increase in cumulative time of SAP < 80% pre-induction value adjusted to gender, age and the Charlson Comorbidity Index was 1.020 (95% CI 1.008–1.035; *P* = 0.003). The coefficients of the model including their uncertainty are listed in Table 2.

**Table 2:** Proportional odds model with time SAP < 80% pre-induction baseline as a hypothesised predictor

Model					
Time SAP < 80% pre-ind + age at surgery + gender + pre-op Charlson Comorbidity Index					
Coefficient	*β*	SE	Odds ratio	95% CI	*P* value*
Intercept 0|1	3.314	2.078			
Intercept 1|2	4.562	2.099			
Intercept 2| ≥ 3	6.939	2.225			
Time SAP < 80% pre-ind	0.02017	0.00681	1.020	1.008–1.035	0.003
Age at surgery	0.06990	0.02591	1.072	1.023–1.134	0.007
Pre-op Charlson CI	− 0.17153	0.10938	0.842	0.676–1.042	0.117
Gender (Female)	− 0.93429	0.74854	0.393	0.085–1.661	0.212

*P value obtained from Wald’s test statistic

### Multivariable analysis with the Comprehensive Complication Index as outcome

Multivariable regression with the Comprehensive Complication index as dependent variable showed that also for this outcome, including cumulative time of SAP < 80% below pre-induction baseline as a covariate for cumulative time of hypotension produced the best fitting model, compared to models where the cumulative time of hypotension covariate was replaced with the other hypotension variables (*P* < 0.001 compared to other models). Adding cumulative time of SAP < 80% pre-induction baseline to the model also significantly improved the goodness-of-fit over the base model (*P* = 0.002).

The null hypothesis that goodness-of-fit of the models containing either the Charlson Comorbidity index or ASA as preoperative comorbidity index was not statistically significantly different was rejected, with the model with the former index showing a better fit (*P* = 0.048).

For every additional minute of SAP < 80% pre-induction baseline, the mean Comprehensive Complication Index increased by 0.057 (95% CI − 0.011–0.126; *P* = 0.102). However, this adjusted increase in mean was not statistically significant (Tables 3 and 4).

**Table 3:** Linear model with time SAP < 80% pre-induction baseline as a hypothesised predictor

Model				
Time SAP < 80% pre-ind + age at surgery + gender + pre-op Charlson Comorbidity Index				
Coefficient	*β*	SE	95% CI	*P* value
Intercept	− 21.754	17.266		
Time MAP < 55 mmHg	0.057	0.034	− 0.011–0.126	0.102
Age at surgery	0.582	0.200	0.181–0.984	0.005
Pre-op Charlson CI	− 1.122	1.002	− 3.140–0.895	0.269
Gender (Female)	−2.518	6.249	−15.103 – 10.069	0.689

*P value obtained from Wald’s test statistic

**Table 4:** Criteria used during the retrospective chart review to identify morbidities occurring during the postoperative period (adapted from that described by Bennett-Guerrero et al. [16])

System	Morbidities
Respiratory	New requirement for supplemental oxygen New requirement for respiratory support
Immunological	Antimicrobial therapy Pyrexia
Renal	Oliguria < 500 mL/24 h Rise in serum creatinine, either ≥ 26.4 μmol/L from baseline, or a greater than 1.5-fold rise from baseline Urinary catheter in situ
Gastrointestinal	Unable to tolerate enteral diet for any reason
Cardiovascular	Tests or therapy for any of: - New myocardial infarction or ischaemia - Hypotension (requiring fluid therapy > 200 mL/h or pharmacological therapy) - Atrial or ventricular arrhythmias - Cardiogenic pulmonary oedema - New thrombotic event requiring anticoagulation
Neurological	New focal neurological deficit, confusion, delirium or coma New postoperative pain requiring parenteral opioids or regional analgesia
Haematological	Requirement for blood product transfusion: - Red cell concentrate - Platelets - Fresh frozen plasma - Prothrombin complex concentrate - Fibrinogen
Wound	Wound dehiscence requiring surgical exploration, or drainage of pus from wound

## Discussion

A key challenge in caring for patients with hip fractures is expediting the corrective surgery while minimising the impact of preoperative morbidity and surgical stress, and thereby reducing the risk of postoperative complications. This objective has been delineated in guidelines from various bodies including the Scottish Intercollegiate Guidelines Network, the National Institute for Health and Clinical Excellence and the Association of Anaesthetists [27–29]. Irish best practice tariff measures for hip fracture care that all patients who are medically fit should have surgery during normal working hours and within 48 h of admission; further, patients with fragility fractures should have routine access to acute orthogeriatric medical support throughout their stay [1]. There is little evidence that generic prehabilitation or preoptimization strategies improve outcomes in hip fracture patients, but selected patients with serious intercurrent illness may benefit from a short period of medical management and stabilisation prior to their operation [30]. As our study was retrospective, our quantification of postoperative morbidities was limited to those identified in the written record by the patients’ care teams during their hospital admission, and our review may therefore underestimate the total morbidity burden of the patient cohort. This may be particularly true for conditions like delirium, which is common in hip fracture patients but can be missed if not sought systematically. The Irish Hip Fracture Database uses broader alternative outcome measurements including cumulative length of stay and the Cumulative Ambulatory Score [1].

Our data demonstrates a correlation between cumulative time of intraoperative hypotension, defined as a fall in SAP to less than 80% pre-induction baseline, and increased postoperative morbidity in this cohort of patients undergoing surgery for hip fracture. Despite a lack of consensus on how best to define hypotension, the Association of Anaesthetists has recommended using this definition as the “least bad” with respect to perioperative care of the elderly [27]. The average age of our patient cohort was 78 years which is slightly younger than the Irish national average for hip fracture patients (81 years in 2018), although it has previously been noted that the Irish cohort appears to be slightly younger than those in other countries [1]. No association between hypotension and postoperative morbidity was evident when the baseline for SAP was taken to be the first blood pressure measurement recorded for each patient on admission with their hip fracture. In our cohort, SAP on admission (most commonly in the emergency department) was significantly higher than the last SAP measurement taken prior to induction of anaesthesia; this likely reflects the fact that patients will have received analgesia and other medical therapies in the period between these two measurements, and blood pressure measurements taken prior to anaesthesia may therefore better reflect a patient’s true baseline prior to their injury than blood pressure measurements in the emergency department. Evidence exists to support the assertion that blood pressure should be maintained as close to baseline as possible. One previous retrospective analysis of intraoperative data has suggested that transgression below a MAP threshold of 55 mmHg, even for short durations, is a sensitive predictor of postoperative acute kidney injury and myocardial injury [15]. Another analysis has suggested that both relative and absolute reductions in MAP may be equally effective [28, 31].

Most studies on anaesthetic management of hip fracture patients have focused on whether anaesthetic technique (typically general versus spinal anaesthesia) affects postoperative mortality, with a clear signal that long-term mortality after hip fracture surgery remains in the order of 5–8% regardless of anaesthetic technique [6, 32]. Recent evidence more commonly supports the use of spinal anaesthesia and regional techniques over general anaesthesia, as they are associated with reduced incidence of intraoperative hypotension [28]. Observational studies have noted high incidence of intraoperative hypotension, despite the heterogeneity of definitions of hypotension used, which is directly associated with increased 30-sday mortality [5]. Combined spinal and general techniques may be associated with precipitous falls in blood pressure [12]. Another cohort study found that hospitals with higher utilisation of neuraxial anaesthesia for hip fracture surgery had lower mortality rates after hip fracture surgery, but that the difference in survival was not attributable to the utilisation of neuraxial anaesthesia itself; instead, this served as a marker for other hospital-level factors such as provider skill level, the degree of preoperative risk stratification and avoiding preventable complications [33]. A recent prospective randomised controlled trial in younger, but higher risk, patients found that patients randomised to receive maintenance of intraoperative arterial blood pressure close to baseline had significantly reduced postoperative major morbidity compared with those randomised to standard intraoperative arterial blood pressure management [13]. Another ongoing multi-centre pilot study aims to assess outcomes in older hip fracture patients (aged more than 70 years) by randomising then between tight intraoperative blood pressure control and standard care [34].

This cohort of frail patients who had sustained hip fractures were at a high risk of organ failure even before surgery, as illustrated by their high Charlson Comorbidity Index (mean 5.7, estimated 10-year survival 2–21%). The present study suggests that the use of invasive blood pressure monitoring is associated with significantly less inadvertent hypotension than non-invasive pressure monitoring in such patients; the Association of Anaesthetists has previously recommended routine use of intraarterial cannulation and transduction for this purpose in elderly patients, particularly for major or emergency surgery [27]. One difficulty that arose during our analysis of the data was how to account for the intermittent nature of non-invasive monitoring of arterial pressure: if a patient had two hypotensive blood pressure readings 3 min apart, is it is more helpful or accurate when quantifying hypotension to count this as a 3-min period of continuous hypotension, or to record it as two separate instances of low blood pressure readings? Our analysis was retrospective, and the use of invasive or non-invasive blood pressure monitoring was non-randomised and non-blinded in our cohort. Further prospective studies are warranted to demonstrate whether invasive monitoring is effective as a means of reducing the duration of hypotension.

Little data is available on what pharmacological agents, and what modes of administration, are most effective in treating hypotension during anaesthesia for hip fracture surgery. Many agents have been very well studied in the obstetric population [35]. In this context, phenylephrine, metaraminol and noradrenaline infusions are all potential candidates for peripheral short-term infusion strategies [36, 37]. Ephedrine can also be used by infusion and is effective, though it may lead to more episodes of dysrhythmia [38]. Phenylephrine has been the agent of choice for treating spinal anaesthesia induced hypotension due to its primary vasoconstrictor action, while ephedrine preferentially acts to improve cardiac output [39]. However, a recent prospective study on the effects of phenylephrine for anaesthesia induced hypotension showed that it was effective at increasing cardiac output in patients who were relatively hypovolemic [36]. Many patients in the hip fracture cohort will have a degree of physiological beta-blockade secondary to age-related autonomic dysfunction, making alpha-1 adrenoceptor agonists the treatment of choice [39, 40]. It is not clear whether hypotension is more reliably prevented in high-risk patients by administering intermittent boluses purely when deemed appropriate by the clinician, or by using a continuous infusion. Studies also focus on the importance of intravascular filling and the role of adequate preoperative loading with either colloid or crystalloid [41].

Finally, the present study examined hypotension only during the intraoperative period, from the induction of anaesthesia until onward transfer of the patient’s care in the postanaesthesia care unit. The role of postoperative hypotension in relation to postoperative morbidity merits closer investigation. Guidelines have previously recommended that all patients with a preoperative estimated mortality in excess of 10% should be admitted to a level 2 or 3 critical care facility after their surgery, where continuous blood pressure monitoring (among other therapies) is available, although in many countries practical resource limitations will not always allow for this [42]. Potential future technological developments in telemetry or wireless monitoring could offer novel solutions to this problem, and remote monitoring of at-risk patients is a prospective area of future investigation.

This study has some important limitations. The sample size of the study is small, particularly with respect to the number of parameters estimated. This significantly affects the estimation precision of the parameters of interest and makes it difficult to determine which cumulative time of hypotension variable is closer associated with postoperative comorbidities. Another limiting factor of the small sample size was the necessity to collapse ordinal variables in the study, limiting its generalisability, which is further affected by the use of a single centre for patient recruitment for the study. Another limitation is the dichotomisation of the blood pressure variables, which negatively affects the power of parameter estimation and limits the ability to include interactions between variables in the prediction models. Finally, the small sample size does not allow for validation of the prediction models on test cohorts, which increases the risk of overfitting and lowers the generalisability of the study’s findings. For these reasons, the study results cannot be treated as more than exploratory.

## Conclusions

This exploratory, single-centre, retrospective analysis of elderly patients undergoing hip fracture repair demonstrated an association between the duration and extent of intraoperative hypotension and increased postoperative morbidity, irrespective of anaesthetic technique. The duration of hypotension while under anaesthesia is a good candidate for further study as a predictor of postoperative complications. Duration of time under anaesthesia where MAP < 55 mmHg may be more predictive of postoperative complications than MAP < 75 mmHg. Defining hypotension as a reduction in SAP below 80% of the baseline immediately prior to induction of anaesthesia may be more predictive than either using the initial SAP on admission to hospital as the baseline or using MAP < 55 mmHg as the definition of hypotension.

The hypothesis that actively maintaining intraoperative blood pressure close to preoperative baseline values will reduce postoperative morbidity, compared with patients receiving standard intraoperative blood pressure management, warrants evaluation in a prospective, randomised controlled trial.
